# Professional regulation and attitudinal issues: constructing the ‘good doctor’ and the ‘bad apple’ through the device of insight

**DOI:** 10.1093/medlaw/fwaf032

**Published:** 2025-08-28

**Authors:** Paula Case

**Affiliations:** Department of Law, School of Law and Social Justice, University of Liverpool, Liverpool, L69 7ZR, United Kingdom

**Keywords:** doctors, fitness, insight, misconduct, professional, regulation

## Abstract

In fitness-to-practise hearings for doctors and other health care professions, the practitioner’s ‘insight’ into their past misconduct or deficient performance takes centre stage in determining outcomes. Although insight does not feature in the statutory framework, it has emerged as a regulatory device used to distinguish the ‘good doctor’ from the ‘bad apple’ who should be excluded from the profession in order to protect the public. This article frames the concept of ‘insight’ in professional discipline as an example of Foucauldian avowal—a ritual of truth-telling which requires the doctor’s full acknowledgement and admission of wrongdoing and a disavowal of their former self. If successful, avowal enables reintegration of the practitioner into the profession’s social order. ‘Insight’ is then tracked as a contested site in doctors’ fitness to practise appeals across a period of more than 25 years, exploring its clinical origins, expansion, and modern application. The accumulated case law confirms insight as a deeply embedded staple of fitness to practise decision-making and a core regulatory strategy for protecting patients, while highlighting that it is inevitably compromised by issues of authenticity.

## I. INTRODUCTION

The fitness to practise machine in healthcare professions regulation^1^ is programmed to (amongst other things) identify and exclude dysfunctional practitioners^2^ in order to keep patients safe. The space afforded by fitness to practise investigations into misconduct and deficient performance is used to closely examine the practitioners’ commitment to professional ideals and their moral ‘character’.^3^ Regulatory bodies construct these assessments from various cues in the practitioner’s reactions and responses to the allegations made against them—a process captured by the language of attitudinal factors and, specifically, ‘insight’ and remediation. While this article concerns the specific context of doctors’ regulation, the conceptual tool of insight has currency across the healthcare professions regulation in the UK,^4^ and any findings herein have relevance to the governance of nurses, dentists, opticians, pharmacists, and many other health care workers.^5^

In the UK, regulators of the healthcare professions are subject to an ‘overarching statutory function’ of ‘protecting the public’, an objective which extends to promoting and maintaining both the health, safety, and wellbeing of the public and public confidence in the medical profession.^6^ When considered ‘genuine’, the practitioner’s performances of insight (which can include acknowledgement and/or admission of wrongdoing or deficiency, remorse, empathy towards those affected, contrition, apology, and remediation) are tightly bound up with regulators’ assessments of the risk posed to the public by returning the practitioner to practice. In *Ibrahim v General Medical Council*, the court regarded the doctor’s ‘incomplete’ insight into his conviction for an offence of domestic violence as indicating a risk of repetition, and in *Sawati v General Medical Council*, the assessment of the doctor’s insight into incidents of dishonesty, including dishonestly amending a patient’s file, was linked with future risk to patients or to public confidence in general.^7^ Successful representations of insight by the accused can therefore mean the difference between repatriation with their profession and expulsion on the grounds of risks to patients or the public.

Utilizing attitudinal issues to identify practitioners who can safely be returned to practice theoretically offers an optimal regulatory strategy for protecting the public—reducing the risks of recurrence while also delivering on economic efficiency by preserving valued social capital, namely, highly skilled, intensively trained healthcare professionals^8^ when safe to do so. It also involves what might be termed a ‘self-administered’ form of regulation. Expressions of insight are incentivized, with the ideal practitioner self-reporting, self-diagnosing, self-sentencing,^9^ and voicing the ‘internalisation’ of regulatory logic, thereby ‘reducing the need for external regulation’.^10^ The insight construct, which sits at the heart of assessments of what is needed for public protection, is also, however, inherently fragile, for while defendants must appear genuine in their expressions of remorse, these behaviours occur in a setting where substantial gains are being offered for a convincing performance of insight.^11^

Clearly, potential exists for this time-hallowed process of avowal in fitness to practise proceedings, through demonstrations of ‘insight’ and ‘remediation’, to be hijacked by false representations, disingenuous declarations of shame and regret, and therefore *not* be accompanied by the desired transformation or reduced risk to patients and the broader public. Rare judicial musings on insight are found in *Bramhall v GMC*, the case of a renowned liver transplant surgeon discovered to have tattooed his initials on two patients’ livers with an argon coagulator. *Bramhall* sparked media attention due to the public confidence import of revelations that a surgeon was covertly initialling the organs of unconscious patients and concerns about the essential character of this conduct: was this an act of ‘hubris’ showing disregard for the dignity of a vulnerable patient or a flourish at the end of a long and stressful procedure posing no risk of physical harm?^12^ The surgeon’s insight into these events was understandably probed, and Collins Rice J urged vigilance by tribunals—expressions of remorse and insight were not to be taken at face value, but should be evidenced and ‘demonstrably interrogated’.^13^

The remainder of this article explores the application and evolution of ‘insight’ in the regulation of doctors, scrutinizing how insight is evidenced and ‘interrogated’ in cases appealed to the courts, with a particular focus on prominent risks of falsehood in the regulatory search for authentic expressions of insight. The place of ‘insight’ in fitness to practise is examined, framing it as an example of Foucault’s ‘avowal’,^14^ and uncovering its layered functions in regulatory strategy. Discussion then turns in Sections III–IV to unpacking findings from a trawl of doctors’ fitness to practise appeals (from 1997 to 2023) where ‘insight’ features, probing the term’s origins, painting a picture of its gradual rise as a contested issue in appeals, and documenting permutations in its use. In addition to a Foucauldian framing, the analysis also draws from an established body of research into ‘remorse’, a central concept in parole decision-making in the USA and Canada.^15^ The remorse literature challenges decision makers by questioning the feasibility of gauging the authenticity of remorse. Authenticity concerns emerge as the central theme of this article’s analysis of insight in misconduct hearings. In Section V, shifts in the regulatory landscape (particularly, the growth of insight training and moves to reduce formal hearings) are observed to pose new threats to the integrity of practitioner avowal.

## II. TELL ME WHO YOU ARE, SO THAT I MAY JUDGE YOU’: FOUCAULT, AVOWAL, AND ITS FUNCTIONS IN HEALTHCARE REGULATION

In Foucauldian terms, ‘avowal’ is a ritual of truth-telling^16^ often demanded from the accused in penal systems in response to allegations.^17^ A great deal of Foucault’s work focused on the workings of penal systems, and his genealogical approach identified a longitudinal shift in focus from defendants’ conduct ‘towards a truth manifested by the whole individual’,^18^ and ‘the soul of the defendant’.^19^ This focus on the internal self is writ large in the regulation of healthcare professionals. A doctor’s ‘fitness’ can be called into question for a seemingly infinite variety of conduct issues, usually taking the form of: (i) ‘deficient performance’ (eg diagnostic errors, poor record keeping, breaching patient confidentiality), (ii) ‘misconduct’ (eg dishonesty (whether covering up clinical errors, CV dishonesty, or fare dodging on the commute to work) or sexually motivated conduct towards patients or unwanted sexual approaches to colleagues), or (iii) ‘criminal conviction or cautions’ (from assaulting patients to domestic violence, driving offences and recently, unlawful environmental protests^20^).^21^ The GMC oversees the fitness to practise of doctors, and, to this end, investigates and ‘prosecutes’ doctors for alleged misconduct and deficient performance before Medical Practitioner Tribunals (MPTs).^22^ Most decisions about whether a doctor ought to be excluded from the profession (temporarily or permanently) are made by the MPTS. Regulatory restrictions on a doctor’s practice (‘sanctions’ such as conditions on practice, suspension or erasure from the register) are applied by MPTS tribunals only when that doctor’s fitness to practise is judged to be ‘impaired’.^23^ In assessing impairment, decision makers focus not solely upon the incident(s) giving rise to the complaint, but rather the doctor’s conduct ‘in the round’, including evidence of the doctor’s performance prior to *and subsequent to* the conduct in question.^24^ Such an assessment involves taking into account the seriousness of the misconduct, but ‘looking forward not back’^25^ towards the extent to which the practitioner has gained insight into their shortcomings, whether the issues raised are potentially remediable, attempts made by the practitioner to remediate their poor practice or misconduct and the risk of recurrence.^26^

Insight (or the lack thereof) is also an important factor in determining (post a determination of ‘impairment’) *which* sanction should be imposed. A persistent lack of insight has long been ‘a pertinent issue’ on the appropriateness of erasure,^27^ and assessment of insight is a key consideration in relation to each of the other sanctions.^28^ Insight is treated as cumulative, with tribunals often referring to it as ‘developing’ or ‘incomplete’,^29^ but there is also a binary dimension, in that a failure to advance evidence on the doctor’s insight supports a conclusion that/presumption that the doctor lacks insight.^30^

Although Foucault’s ‘avowal’ is often equated simply with ‘confession’,^31^ his ‘grand tour’ of penal systems in *Wrong-doing, Truth-telling*^32^ depicts avowal as comprising two components. Avowal is both: (i) an admission or acknowledgement^33^ (especially of wrong-doing), involving recognition of guilt and being ‘the first pledge of the punitive pact’, to receive sanction and to participate in the corrective process^34^ and (ii) a disavowal of the accused’s former self, and therefore an act of ‘self-constitution’.^35^ Ideally, there will be evidence of self-transformation, a ‘personal epiphany’ convincing the tribunal that the self that committed the transgression is separate and distinct^36^ from the new post-offence self (or the retrieved pre-offence self); a splitting of the self into the part that committed the transgression, and the part expressing the ‘true’ self who rejects the conduct as morally unacceptable.^37^ In the review hearing of *Arunachalam*, the doctor accused of predatory harassment of two junior colleagues presented as a ‘changed man’.^38^ The doctor’s castigation of his earlier behaviour as ‘appalling’, ‘pathetic’, and ‘selfish’^39^ represented both current acceptance of the ideals of the good doctor and a rejection of the past self. The avowal is therefore a ‘double subordination … to an authority and to a regime of truth’.^40^

Avowal thus ‘helps to bestow a sort of self-identity that falls within contextually understood boundaries’.^41^ However, in conceptualizing self-constitution as a key part of avowal, Foucault suggests that the transformed identity is not just imposed but is also actively created by the subject. The avowal therefore contains an implicit promise by the registrant ‘to be what he affirms himself to be, precisely because he is just that’.^42^ Avowal therefore assumes that at the very least, the doctor believes in the truth of their expressions of insight and remediation, that the doctor is indeed a reformed character and that the avowal in its expressions of remorse, apology, understanding, etc., is ‘genuine’. However, there are many threats to judging authenticity, which themselves represent vulnerabilities in the overall regulatory scheme and regulators’ capacity to protect patients. Some particular examples (detailed below) are explored in Section V.

### A. Insight v remorse

‘Insight’ occupies a very similar space in regulatory decision-making to the concept of ‘remorse’ used in some parole/criminal justice contexts, particularly in the USA and Canada.^43^ These concepts clearly overlap substantially, but there is some conceptual confusion. In the case of *Lingam*, the doctor’s remorse about their ‘cavalier’ prescribing was not, without more, evidence of insight,^44^ but compare *Bramhall* where insight is regarded as a step towards remorse,^45^ and *Williams* where insight and remorse were consecutive steps on the way to ‘redemption’.^46^ One area of broad agreement is that these terms have purportedly distinct temporal dimensions, with ‘remorse’ generally treated as located in defendants’ emotional expressions about *past* conduct, and ‘insight’^47^ being a subjectively held state, and objectively judged to reflect the risk of *future* repetition.

Weisman’s study of remorse in ‘Being and Doing’ bridged the sociology of emotion^48^ and legal scholarship, offering a detailed socio-legal analysis of modern Canadian court judgments.^49^ While remorse literature generally, and Weisman’s study in particular, can be viewed as a conversation in law and emotion scholarship,^50^ it also bears traces of Foucauldian thought, in that a central theme is the notion of a ‘remedial exchange’^51^ in the aftermath of the defendant’s transgression from society’s moral code. Expressions of remorse (which, within certain parameters, are frequently accepted as a form of mitigation) ‘reward’ offenders with a reduced sentence or early/discretionary parole.^52^ In this sense, they are a ‘contract of truth’ which builds from the ‘implicit pact on which is founded the sovereignty of the institution that judges’,^53^ and agrees on a particular interpretation of the misconduct or deficiency.

This study builds sideways from Weisman’s work, examining judicial engagement with ‘insight’ (rather than ‘remorse’), in the specific context of appeals from doctor misconduct hearings in the UK’s Medical Practitioner Tribunals. Appeals against a fitness to practise (‘ftp’) decision by a tribunal can be launched by either the registrant (doctor) or by a regulatory body,^54^ and the resulting judgments provide a rich source of data on judicial oversight of expressions of insight. Section III outlines the dataset of cases used to examine the contours of insight, exploring preliminary matters such as tracking the increased frequency with which insight has been referenced in appeals and making some observations about its origins and the conceptual fuzziness associated with its use.

## III. JUDICIAL ‘OVERSIGHT OF INSIGHT’

### A. Methodology: tracking the intensity of ‘Insight’ referencing in doctors’ fitness to practise appeals

**Figure 1.**
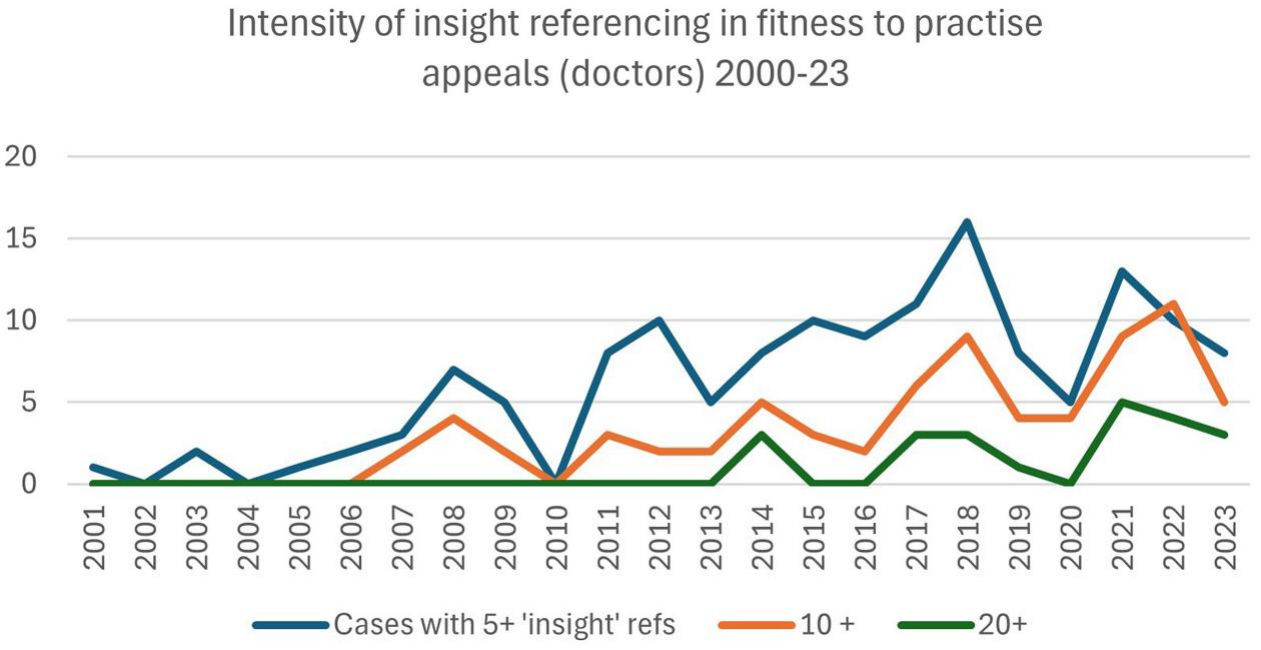
Intensity of ‘Insight’ referencing in GMC appeal cases from 2000 to 2023.

The findings below represent the results of a *Westlaw*-powered case trawl for fitness to practise appeal judgments which engaged with the concept of ‘insight’ where the GMC was appellant or respondent.^55^ A total of 333 fitness to practice judgements were returned, spanning 26 years,^56^ with early cases including only fleeting references to ‘insight’, rather than any elaboration or interrogation of it as a concept. Cases with 5+ references to ‘insight’ (and also 10+ references and 20+ references) were tracked, in order to map levels of engagement with insight in these cases from 2000 to 2023. The results, presented in Fig. 1, chart the increase in judicial engagement with ‘insight’ over time. Although this growth in usage is non-linear,^57^ it is consistent with a steady rise of ‘insight’ as a site of contention in fitness to practise cases.

‘Insight’ was then tracked through the returned cases to interrogate its origins, expansion, and judicial oversight of its application, including judicial assessments of whether demonstrations of insight and remediation were genuine (to the extent that these are visible). While studying the use of insight in tribunal decisions would also be bound to yield interesting results, only one year’s worth of these first instance decisions is publicly available at any given time. Such a study would therefore generate a ‘snapshot’ rather than a longitudinal view. Examining judicial engagement with insight in professional discipline appeals over more than 20 years enabled the researcher to explore the development of insight jurisprudence over time and changes in its use.

### B. Early references: transplanting clinical concepts?

The earliest mentions of ‘insight’ in the author’s dataset appear in the 1997 case of *Stefan v General Medical Council* relating to fitness to practise concerns on mental health grounds and the doctor’s refusal to engage with a psychiatrist.^58^ Subsequent references to insight in the early 2000s appear in just a handful of cases,^59^ but like *Stefan*, these were usually appeals from what was then the GMC’s ‘Health Committee’. Evidence was led by psychiatrists that the doctor lacked insight into their illness (eg alcohol-related or a bipolar diagnosis),^60^ and therefore there were real concerns for the doctor’s ability to practise medicine. The longstanding clinical concept of ‘insight’ is still used in the context of patients with mental health disorders (usually to mean self-awareness of illness).^61^ It seems likely, therefore, that the origins of ‘insight’ in medical regulation are partly clinical in nature, being mainly found in psychiatric evidence, before being ‘upcycled’ for broader use as a risk assessment tool in later cases.

It is unsurprising that a system, at that time still dominated by members of the medical profession,^62^ borrowed from clinical devices in its regulatory work. It may be worth noting that the clinical use of ‘insight’ has a long and controversial history,^63^ rooted in an era when the process of ‘diagnosis was assumed to be infallible and the patients’ perspective on their experience was marginalised’.^64^ It is often burdened with problematic expectations of ‘compliance’^65^ (ie that an insightful patient will be compliant with their treatment regime and accept the language of the diagnosis and assessment), and this ‘compliance connection’ carries through to professional regulation: insight is often treated as an indicator of a doctor who is likely to be compliant with regulatory requirements in the future.^66^

Beyond mental health cases, references to insight between 1997 and 2007 are fleeting, but converge in their concern with a doctor’s lack of insight into the gravity of their misconduct.^67^ These patchy beginnings are followed by steady growth and expansion of the concept, and whether the focus is on cases with 5 references to insight or more, 10 or more or 20 plus, there is a general trend of increased engagement with the concept.^68^ Many mentions of insight in the judgments are in fact excerpts from the *Sanctions Guidance (SG)*^69^ used by tribunals when making fitness to practise decisions.^70^ The guidance accords insight centre-stage, making 26 references to ‘insight’. It appears as an undefined umbrella term, encompassing a number of overlapping ideas,^71^ to include: ‘admission, apologies, making efforts to prevent behaviour recurring, or correcting deficiencies in performance … ’.^72^ Its importance is underlined by its placement at the top of the list of ‘mitigating factors’,^73^ with the ‘lack of insight’ headlining the list of ‘aggravating factors’.^74^ The GMC-authored guidance has clearly been instrumental in embedding insight in decision-making, and the GMC itself has been an active participant in shaping professional discipline in a way which has been endorsed, expanded and adjusted by the courts. Further research would be necessary to explore the intricacies of that process.

Now, examining the degree of a doctor’s insight is often an integral part of tribunal hearings. However, judicial perspectives on ‘insight’ as a regulatory tool are varied, ranging from scepticism (castigating it as ‘jargon’,^75^) to the close scrutiny recommended in *Bramhall* with insight being evidenced and ‘demonstrably interrogated’.^76^ This examination of regulatory decision-making through the lens of appeal judgments, underscores the central role that insight plays in professional governance. The term is, however, absent from the statutory framework, it lacks an authoritative definition, and has not been the subject of detailed academic analysis in its own right.

### C. The ‘Genuine Insight’ paradox (or the perversity of self-sentencing)

Oddly, despite giving detailed coverage of issues of mitigation and aggravation, the *Sanctions Guidance* for tribunals does *not* reference the need for expressions of insight to be assessed as ‘genuine’.^77^ There are, however, markers of an assumption in the appeal cases that expressions of insight, remorse/regret and apology should be ‘genuine’, as in ‘other-focused’, rather than contrived or performed out of self-interest. Tribunal and court judgments often use the ‘genuine’ descriptor, for example, that expressions of insight were ‘genuine’,^78^ that the purpose of suspension is to facilitate the development of genuine insight,^79^ or being dismissive of the doctor’s representations on authenticity grounds (‘his apology had not appeared to the tribunal to be genuine’^80^). The common, routinized features of insight mean it is possible for defendants to prepare a ‘redemption script’.^81^ However, the formulization of redemption, in and of itself, generates problems for authenticity. In *Motala v GMC*, the doctor unwittingly gave the impression that he regarded demonstrating insight as a ‘box-ticking exercise’ and therefore the genuineness of his claims was doubted.^82^ Defendants must therefore balance their response somewhere between adherence to the formula and not appearing to be following a script.^83^

Weisman, Zhong and Young, and Chimowitz each highlighted that remorse as a classifier/tool in judicial and quasi-judicial contexts is stymied by concerns around genuineness.^84^ So little is known about how remorse is assessed^85^ (and how much it is tainted by implicit bias^86^), and also whether assessors can reliably distinguish genuine remorse from fakery.^87^ Remorse research suggests variance in judicial confidence as to the correct identification of true remorse—for some, it is an impossible feat, some defendants are good liars,^88^ whereas other judges happily rely on their intuition in making these calls.^89^ Moreover, Weisman observed the paradox that while defendants must appear genuine in their expressions of remorse, these expressions take place in a ‘context of suspicion’.^90^ In other words, the substantial incentives posed by the criminal justice system to express remorse ‘automatically calls the sincerity of the remorse into question’.^91^ In the fitness to practise domain too there are sceptics.^92^ Doubts as to the reliability of judgements of the authenticity of remorse raise questions about the integrity of a core element of fitness to practise regulation. Given this ‘socio-legal puzzle’^93^ of requiring genuine remorse or insight whilst not being able to know the truth of the defendant’s internal state, there are bound to be vulnerabilities in the regulatory framework where the risks of false positives and false negatives are enhanced, some of which are explored in Section V below.

## IV. THE ROLE OF INSIGHT IN DOCTORS’ AVOWALS: ACKNOWLEDGEMENT AND DISAVOWAL

### A. The ‘First Pledge’: admission/acknowledgment

Mirroring Foucault’s avowal outlined in Section II, insight requires doctors to make an admission or acknowledgement of wrongdoing as a first step.^94^ The cases gathered here demonstrate that this entails (usually) both an admission of what occurred and an explicit understanding of why it happened and the broader impact of falling short of the profession’s standards. As to this ‘broader impact’, judicial engagement with insight points to an essential ‘other regarding’ element, whether empathy or at least understanding of the effect of the misconduct/deficiency on others.^95^ In *Bramhall*, Collins Rice J regarded as essential the ‘demonstration of a degree of *empathetic identification with the perspective of others*’, elaborating that ‘others’ included ‘victims, professional colleagues, the public (including other patients … , actual and potential)’.^96^ Self-serving narratives or ‘chagrin at the personal consequences of public exposure and regulatory and criminal justice action’ did not qualify as true insight.^97^ In *Mok*, the subject’s insight required further development, as the doctor had tended to present himself as the victim in his reflective work rather than acknowledging the impact of the alleged non-consensual sexual acts towards his then partner.^98^

A more prominent theme in the cases, however, is that the defendant’s insight must express an understanding of the causes of their conduct/deficiency.^99^ In *Sawati v GMC* (the case identified as having the highest number of ‘insight’ mentions^100^), insight was summarized as ‘an acknowledgement and appreciation of a failing, its magnitude, and its consequences for others’, regarded as ‘essential for that failing to be properly understood, addressed and eliminated for the future’.^101^ In *Khetyar*, an even more demanding version of insight was evident, requiring that the ‘motivations and triggers for the misconduct [should] be identified and understood’.^102^

For the purposes of both the doctor’s understanding of motives/causes *and* understanding the consequences of the misconduct, that understanding must reflect regulatory ‘logic’.^103^ Regulatory logic frequently equates an understanding of causes with acceptance of *full* personal responsibility (or ‘unconditional agency’ as Weisman called it) for their deficient or wrongful conduct.^104^ Acceptance of the cause(s) of the misconduct should be attributed to ‘personal deficit’ rather than ‘blaming’ others, situational factors or social structures.^105^ A doctor who sought to blame his deceased wife for forwarding a misleading CV to a potential employer was therefore given short shrift,^106^ and a doctor whose lie implicated others only compounded their dishonesty.^107^ Insight requires that defendants do not ‘minimise the gravity’ of their conduct.^108^ A doctor whose clinical performance has been found wanting must ‘fully acknowledge … why past professional performance was deficient’.^109^ In contrast, a doctor guilty of assault arising out of a road rage incident who continued to ‘minimise the gravity’ of their offences had not reached the desired threshold of acknowledgement and therefore insight.^110^

This means that while doctors may disagree with the regulator’s view of the seriousness of their conduct, or the relevance of private conduct for professional purposes, such disagreement may result in findings of a lack of insight. So in *MXM*, the doctor’s assertion that his sexual activity with a partner at the GP practice where he worked and posting of sexual videos online were ‘removed from the practice of medicine’ was treated as indicative of a lack of insight.^111^ The regulator takes a broad view of ‘risk’, extending to conduct outside the practice of medicine when regarded as posing a risk to public confidence in the profession. In *Nkomo’*s case, insight was precluded by a refusal to recognize the impact of fraud convictions for evading child support payments on ‘the reputation of the profession as a whole’.^112^ These cases represent conflict regarding the proper scope of professional regulation, also clearly visible in the case of Dr Benn, a General Practitioner facing allegations that her involvement in peaceful environmental protest with Just Stop Oil in breach of an injunction required restrictions on her ability to practice medicine. Revocation of her suspension from practice required a commitment to avoid similar conduct in the future and a demonstration of insight into ‘the importance of compliance with the law’ as a doctor.^113^ This general need to accept regulatory logic in order to satisfy the demands of insight creates particular tension in the ‘rejected defence’ cases, discussed at the end of Section V.

### B. Disavowal and self-constitution: good doctor or bad apple?

As above, in rejecting and distinguishing the past self, the avowal is an act of ‘self-constitution’, which further implies taking on a circumscribed identity.^114^ In broad terms, the most significant predetermined categories in the fitness-to-practise domain are those of ‘good doctor’^115^ and the ‘bad apple’.^116^ Reflecting on the bad apple/good doctor metaphor, Searle and others’ analysis of over 6000 fitness to practise determinations across three professions advanced three explanatory models for misconduct: the ‘bad apple’ (flawed individual), ‘corrupted barrels’ (social learning of deviant norms from others in the workplace) and ‘poor cellars’ (situational/environmental factors overwhelming the individual).^117^ Their work acknowledges the difference between ‘bad apple’ and ‘others’ as of key importance for regulators, informing the distinction between those who need to be permanently removed from the register, and those ‘who can be reformed’.^118^ In this respect, expressions of insight function as a blunt profiling or diagnostic tool. The ‘good doctor’ can successfully convince a tribunal of their insight and remediation, and is judged, despite their prior misconduct or deficient performance, to present a low risk of repetition.^119^ The ‘bad apple’ represents an unacceptable ongoing risk to the public, condemned not just by their misconduct, but by their compounding failure to demonstrate sufficient insight and remediation.^120^ In some of these cases, ‘serious attitudinal issues’ are identified,^121^ such as being combative, aggressive or blaming others,^122^ even blaming the regulator’s ‘lack of insight’ for their predicament.^123^ Encased within this dichotomy of good doctor/bad apple, are transitional categories of ‘developing insight’, of being *en route* somewhere between these polarized identities.^124^ Here, tribunals identify the *potential* to reach the category of good doctor, and schedule review hearings to monitor progress.^125^

Contrasting two broadly similar sexual misconduct cases highlights the potency of well-executed avowal via insight and remediation and its role in constituting identity. The doctor in *Mehta* was guilty of sexually motivated conduct towards a junior colleague. Inviting her to look at his powerpoints in his office, he asked personal questions and kissed her neck and shoulder. *Mehta’s* redemption is an excellent example of apology by ‘mortification’.^126^ His demonstrations of ‘exceptional’ insight, remorse, and attempts at remediation (running staff seminars educating staff on boundaries, specifically based on his own misconduct, and encouraging junior doctors to speak up), meant his fitness to practise was found not to be impaired and no sanction was imposed, the tribunal being satisfied that there was no risk of repetition; ‘He had held himself out as an example from which other doctors might learn’.^127^ The judgment documents that despite an initial lack of candour, a ‘gradual’ process of ‘change’ in him occurred culminating in accepting the complainant’s truth.^128^ In *Hanson*,^129^ on broadly similar facts (grabbing a nurse by the hips during a nightshift when alone together during a nightshift and asking her personal questions), the doctor did not engage with the process, had not ‘acknowledged his wrongdoing or expressed any remorse or regret’,^130^ did not attend, was unrepresented, provided no evidence of insight into his conduct and was erased from the register. Clearly, registrants cannot opt out of categorization by not participating, for non-engagement and unresponsiveness to regulatory prompts will invite a conclusion that the registrant lacks insight into their misconduct, and therefore represents a continuing risk to the public.^131^ Convincing evidence of insight and remediation can be pivotal for the individual practitioner’s professional future, but it is also fundamental to the tribunals’ delivery of its statutory objective of ‘protecting the public’.^132^

## V. PROBLEMS OF AUTHENTICITY IN DOCTORS’ INSIGHT: RISKS OF ‘FALSE NEGATIVES’ AND ‘FALSE POSITIVES’

As fitness to practice rhetoric frames the insight inquiry as a tool of public protection deployed to gauge the risks of returning the doctor to practice, it is vital that prosecutors, adjudicators, and the courts are attuned to the risks of false negatives and false positives in those assessments. Analysis now returns to ‘authenticity’, exploring five risk factors for false positives and false negatives in the assessment of ‘genuine’ insight: (i) over- individualization of responsibility, resulting in the occlusion of systemic factors; (ii) the ‘hidden curriculum’ of insight which can disadvantage some defendants; (iii) outsourcing of insight, through the commercial availability of insight training; (iv) ‘accepted outcomes’—proposals to divert decision-making away from extended formal hearings to a form of summary process; and (v) the contested relationship between insight and a doctor’s denial of misconduct allegations.

### A. Individualizing blame and sidelining systemic factors

Potential for false negatives, that is, findings of an absence of insight and a failure to return a safe doctor to practice, flows from the ‘individualising thrust’^133^ of insight, an approach which risks masking or overlooking other pertinent factors in the production of error or misconduct. A troubling determination by decision makers to assess personal fault shorn of its broader context is found in *Bawa-Garba v GMC*, a case concerning a doctor’s conduct in connection with a young patient’s death from sepsis.^134^ Counsel for the GMC had argued for a complete separation of individual and systemic culpability. The GMC maintained that:

systemic failures on the part of the Trust (including IT delays in blood test results being available, heavy reliance on agency nurses who had failed to communicate key matters and working 12-13 hours (a double shift) without any breaks) were irrelevant to impairment and sanction, because what was in issue was the personal failings of the doctor and not those of the Trust as an institution.^135^

However, excluding consideration of broader issues creates problematic tensions with wider acknowledgements that systemic factors (staff shortages, resources, poor management) may be part of the narrative of the doctor’s misconduct or deficiency.^136^ Unstinting emphasis on the individual can mean that insight is treated as incomplete because the doctor continues to blame external, systemic factors for the harms caused. In *Veeravalli v GMC*, the doctor’s blaming of ‘staff shortages, miscommunication, rota changes and confusion amongst the junior medical and nursing staff and other human factors’ for the circumstances regarding the death of the complainant’s baby during childbirth was an obstacle to his insight.^137^ Conversely, the fact that a doctor *does not* blame their workload for the alleged deficiency or misconduct may work in their favour when it comes to expressions of insight.^138^ Incentivising a particular format of insight risks suppressing some of those systemic explanations so that they do not surface at all in the practitioner’s fitness to practise hearing.

That said, there are indicators that tribunals can be sympathetic to systemic and cultural factors (such as a ‘difficult atmosphere and the shocking and old-fashioned culture at the hospital’), recognizing that they can contribute to allegations being brought against a doctor, even if they are not acknowledged in the process of capturing insight.^139^ There are also hints in the cases that ‘isolated’ acts of dishonesty in the context of challenging ‘front-line’ situations involving direct interaction with patients may attract some leniency.^140^ Post-hearing paperwork also now includes a form designed to capture any systemic concerns raised by the hearing, which can then be picked up with the relevant healthcare provider.^141^

However, the fact remains that defendants facing allegations will often have an uncomfortable choice: (i) fully internalizing and reproducing regulatory logic in order to satisfy tribunals of their insight, potentially obscuring competing truths about external contributory factors and missing opportunities to better protect the public; or (ii) refusing to internalize regulatory logic and adhering to a ‘resistance’^142^ narrative. The doctor’s resistance may be motivated by either an unwillingness to be silenced on external factors or, alternatively, a defensive posture, adopted not because of an internal inability to reflect on personal shortcomings but a fear of wider ramifications. Fitness to practice sits within a broader matrix of regulation, and a fault-based redress system.^143^ While ‘apologies’ are protected in the sense of not being ‘treated as admissions of negligence or breach of duty’,^144^ the relevant statutory provision does not extend to demonstrations of ‘insight’ in fitness to practise proceedings. From a doctor’s perspective, complete acceptance of responsibility in order to fulfil the requirements of insight could expose them to civil liability towards any patient whose care is implicated by the matters referred to in the allegations.

### B. Cultural bias and a ‘Hidden Curriculum’^145^ of insight?

Certain groups of doctors will find it easier to access the tools required to successfully demonstrate insight, creating risks that decision makers will arrive at false negative conclusions in respect of doctors without that access. As the cases confirm, avoiding exclusion through successful avowal can require appreciation of the rules, including that *early* and *full* acceptance of responsibility are often required for true insight,^146^ that the impact on patients, colleagues, and public confidence in the profession should be understood and made explicit^147^ and that non-engagement with the process or non-attendance at a hearing and having no legal representation is to invite erasure^148^ or ‘professional suicide’.^149^

#### 1. Language and cultural factors

Tribunals are expressly alerted to the fact that expressions (and assessments) of insight^150^ can be shaped by language and cultural norms.^151^ As with remorse, ‘cultural and social norms and display rules’ (including word choice), can impact how a doctor’s reflections are received and perceptions of the quality of insight demonstrated.^152^ In *GMC v Ahmed* (the doctor here being unrepresented), the court rejected criticisms of the defendant’s use of the word ‘perceived’: ‘I am sorry I was perceived to lack insight into its seriousness when I was explaining my reasons for undertaking such an action’. And ‘I am sorry that during the MPTS hearing I was perceived to view women inappropriately. … I do not feel I have a deep-seated attitudinal issue toward females and I do not see female patients as sexual partners, I am sorry if I was perceived this way.’^153^ The appeal court noted the need to avoid too much focus on nuance, given that English was not the doctor’s first language. In the broader context of many other statements of reflection, the doctor’s wording was not enough to overturn the tribunal’s 2-month suspension for allegations including making sexually motivated ‘friend’ requests on Facebook in respect of two patients.^154^ While in this instance, nuances in language did not trigger adverse findings against the doctor, the prosecutor’s attempt to attach great significance to word choice highlights the problems that these subtleties can cause.

Long-held suspicions that ethnicity distorts fitness to practise determinations have met with conflicting evidence. GMC commissioned research into hearing outcomes from 2012 to 2017, suggesting that the severity of sanctions was *not* associated with personal characteristics (such as international qualification or race), but *was* associated with levels of engagement with the hearing (including attendance) and legal representation.^155^ While this acknowledges that doctors without legal representation (wherever they qualified) face a higher risk of being struck off,^156^ it glosses over the fact that doctors who trained overseas are probably less likely to have access to reliable advice on these issues or legal representation.^157^ The distinction between having indemnity to cover negligence claims and having indemnity to cover defence costs in fitness to practise cases is not always well understood,^158^ and doctors who qualified and/or trained outside of the UK are less likely to have been inducted in the importance of having professional indemnity^159^ (and may not have experienced revalidation, ‘which encourages doctors to reflect on things that go wrong to gain insight’^160^).

For some doctors with overseas training and/or qualifications, the performance rules surrounding the phrasing and format of avowal may represent a ‘hidden curriculum’ which they have been excluded from.^161^ Having robust professional support networks^162^ can steer doctors in the right direction, as can being a member of a good defence organization,^163^ and good legal representation, all of which are less likely for doctors qualifying overseas. But what would help? Important work is emerging on the barriers to performing implied standards of ‘professionalism’ as experienced by marginalized groups,^164^ but work could be done to level out access to support and education. Although offering no guarantees as to the eradication of such issues, diversity in tribunal membership would be a relevant step forward and could infuse tribunals with increased awareness of cases where cultural bias is at play. What appears to be a high proportion of doctors registered to practice in the UK reporting to be from minority ethnic groups (50.1 per cent of UK registered doctors being non-white or not specified),^165^ is far from being matched by tribunal membership (only 23 per cent of tribunal members were identified as from minority ethnic groups as of September 2023).^166^ Work is needed here to redress the balance.

### C. Redemption scripts and outsourcing the curation of insight

Insight is now for sale. Culturally shared understandings regarding the profession’s rules and parameters of demonstrating insight have resulted in the emergence of a commercial market in insight. There are instances of psychologists or psychiatrists being commissioned in part to assist accused doctors in their demonstration of insight.^167^ But there are also now consultancy services, advertised to those facing fitness to practise allegations, including online or ‘in person’ courses and one-to-one tuition. Some provision is specifically focused on the demonstration of insight and remediation for the purposes of a favourable outcome in a fitness to practise hearing, but this is complemented by a broader menu of options, including courses on sexual boundaries, the importance of candour, honesty, etc.^168^ Returning to Foucault’s words, ‘tell me who you are so that I may judge you’, questions arise regarding whether these paid-for services further complicate assessing authenticity. Does the use of these commercial routes to honing insight obscure the knowing of the person? Do courses teach individuals to be more reflective or just more savvy? Does the tuition itself become constitutive, reshaping the person? And does any transformative impact outlive the hearing?

The judgments reveal that it is common practice for doctors to rely on the completion of such courses to satisfy a tribunal as to insight and readiness for a return to safe practice. There is evidence of some limited judicial scrutiny in these cases.^169^ While the tribunals and courts do not generally question this practice as a matter of principle, there are signs that the courts can be sceptical of the utility of taking a course (on its own) to demonstrate insight. In *Somuah-Boateng*, the MPT had been unconvinced that a three-day course on ‘sexual boundaries’ demonstrated any evidence of insight into his sexual relationship with a vulnerable patient.^170^ Furthermore, in *Armstrong*, the defendant’s attendance at courses in medical ethics and medical professionalism and engagement with an ‘extensive reading list’ was regarded as having ‘no material bearing’ on her insight, and the ‘Tribunal [had] failed to explain’ why it had taken a contrary view.^171^ Courses do, however, seem to be valued by tribunals, particularly in cases where the practitioner’s suspension from practice disables them from demonstrating insight through their work.^172^

Given that assessments of insight are so pivotal to decision-making, and use of these services seems widespread, specific scrutiny of that provision is needed. Two particular concerns are raised here. First, the quality of insight consultancy is variable, and there is no accreditation scheme in place. Secondly, there are questions of whether we should be concerned that some of these services may effectively ‘coach’ the defendant in the ‘correct’ responses, thereby masking continued attitudinal issues. On quality, there are clearly some extremely high-calibre insight services on offer, nurturing the defendant doctor in their pursuit of genuine reflection and redemption. However, in the online environment in particular, there are courses at the other end of the spectrum. Some of these were sampled by the author. In one instance, the text, visual content, and voiceover appeared to be largely Artificial Intelligence (AI) generated and of little obvious practical value. In another, a ‘one-hour’ course with extremely repetitive and unchallenging content was doable in around 10 minutes, including finishing the end-of-course quiz and printing off the certificate, yet this provider’s courses are cited in a number of fitness to practise hearings. Regulators need to take an active interest in the provenance of these courses so that they can meaningfully distinguish between worthwhile provision with assessed outcomes and that which merely provides lip service to the values underpinning fitness to practise. The former could be harnessed to play a part in levelling up in cases which might otherwise be impacted by a hidden insight curriculum, whereas the latter should be disregarded.

On coaching, lawyers will know that professional ethics draws a line between ‘witness preparation’ (or familiarization) and ‘coaching’, the latter being prohibited.^173^ Coaching contaminates the fact-finding process,^174^ but it is unclear at what point support and facilitation of reflection and ethics training might become a type of coaching, and to what extent coaching in this context is objectionable, if ever.^175^ The rules against legal representatives coaching defendants would not be applicable, as the service provider here (although sometimes led by lawyers) is separate from the process of legal representation. Notwithstanding this separation, there are still ethical issues at stake. A provider who nudges the doctor to drink at the pool of reflection and facilitates the professional’s transformation has not compromised the integrity of the insight process, whereas a provider who shapes the doctor’s testimony and conceals their true nature has contaminated proceedings. The hope would be that cross-examination at the hearing would be capable of distinguishing between the parroting of learned responses and sincere attempts at redemption. Unfortunately (as per below), in future, expressions of insight are increasingly likely to be reduced to a written exercise with little opportunity for cross-examination.

### D. Accepted outcomes and diminishing spaces for embodied insight

Foucault envisaged that for the performance of avowal to be effective, it should be a public, verbalized affair^176^—it should not happen in private^177^ and must be ‘certified’.^178^ As Rossmanith observes ‘live bodies facilitate truth telling’^179^ (or at least truth detection). There is, however, no rule requiring doctors to attend their fitness to practise hearing in person. And while expressions of insight can be written (rather than presented in the flesh and subject to cross-examination),^180^ Justice Yip in *Yusuff* noted that it was more ‘difficult’ to assess issues of remorse and insight on paper.^181^

Whether presented in person or paper form, avowal in fitness to practise retains a ‘public’ element. Hearings are generally open to the public,^182^ and some of the details of the doctor’s avowal become embedded in the minutes of the hearing, which are then posted online in the aftermath of the decision.^183^ Even after removal from the MPTS webpages, they remain appended to the defendant’s entry on the medical register held by the GMC, and, although less visible, are viewable by any member of the public who decides to look for them.^184^ The courts certainly appear to assume a public audience for the minutes of the tribunal decision, referencing ‘the reader’ (of the tribunal judgment) being left in an unsatisfactory limbo due to inadequacies of reasoning.^185^

Proposed reforms will see a reduction in both the public and verbal aspects of fitness to practice assessments. Fewer cases will proceed to fitness to practise hearings, with some being diverted to a more cost-efficient, less adversarial ‘accepted outcome’ route, where decisions will be taken by ‘case examiners’ rather than a panel.^186^ If accepted outcomes become the norm in professional discipline, the visibility and transparency of avowals will be much reduced. The PSA has warned that ‘panels are likely to be better placed to make a robust assessment of insight’, casting doubt on the use of a summary procedure to do the same.^187^

Difficulties of assessing insight posed by necessarily increased reliance on written statements of insight due to this summary process will be further exacerbated by the now widespread access to AI platforms. ChatGPT, Open AI, and similar can be utilized to generate convincing expressions of remorse and apology,^188^ enabling further ‘outsourcing’^189^ of emotional responses to a regulator’s attention. A decision maker’s awareness that AI has been used more than moderately in the production of a redemption script is likely to substantially downgrade its perceived authenticity,^190^ and may even compound concerns about the defendant’s character. However, detection of such use is unlikely (unless the defendant specifically discloses it). Paradoxically, these AI concerns strengthen the argument for embodied insight, precisely at a time when formal hearings are likely to decline. They also strengthen the argument for a focus on *how* the defendant has managed their responses and any deeds of redemption, rather than what is said by way of insight.

### *E*. *The rejected defence: avowal without admission?*

The first step to showing genuine insight is *usually* for the doctor to acknowledge what they have done wrong. This is key to avowal and Foucault regards resistance as incompatible with avowal.^191^ Therefore the fact of continued denial and the demonstration of insight into wrongdoing may appear to be mutually exclusive. Older cases in this dataset, such as *Pillai v GMC*, treated continued denial as a lack of insight,^192^ indicating a risk of false negatives by tribunals ignoring the possibility that the reason for the denial was unconnected with the genuineness of insight, but was a legitimate rejection of the tribunal’s findings. Instances of this issue are known as the ‘rejected defence’ cases, because the question is, if a doctor’s defence has been to deny the allegation of misconduct throughout the investigation (and even after a tribunal’s finding that the misconduct is proven), is that denial to be equated with a lack of insight into their conduct?

*Awan v GMC* neatly illustrates some of the intricacies here. Dr Awan had been found guilty of sexually motivated conduct towards someone he had believed was a minor. He defended the fitness to practise allegations of sexual misconduct, claiming that he had known that ‘SophiaSheff’ (whom he met in an online chatroom) was in fact a police officer and not a 13-year-old girl, and had been ‘playing along’. The GMC argued that his ‘incredible’ defence, and persistence in it, even *after* the tribunal had found the facts proved against him, demonstrated a lack of insight.^193^ The court observed, however, that it was expecting too much of doctors to expect them to undergo a ‘Damascene conversion’, changing their narrative once found guilty. If demonstrating insight required a doctor to cease denying their misconduct, they would be compromising their right of appeal (where they may want to argue they did not do what was alleged, and that the tribunal was therefore wrong in its conclusion). Appearing to endorse what might in other contexts be regarded as a ‘non-apology’, the court said it may be acceptable for the doctor to show insight by saying, in effect, ‘I am sorry if what I did/said caused offence’.

The modern approach is therefore more nuanced than that expressed in *Pillai*. Denial at the outset of the case, and continued denial in the face of a tribunal decision that the misconduct has been proved, remains highly relevant to impairment and sanction, as it may speak to ‘attitudinal factors’, but denial is not determinative. This change in position is more than theoretical, with some doctors clearly having returned to practice, despite their continuing denial of the original allegations.^194^ A tailored approach is preferable (albeit messier) than the position set out in *Pillai*; otherwise, the system relies on a flawed assumption that a finding by the MPT of dishonesty or other allegation must be ‘correct’, leaving no room for doubt, and the registrant’s right to a fair trial is heavily compromised. This later position also suggests the need to qualify Foucault’s model of avowal in this context, for avowal appears possible with a degree of self-constitution/transformation, but absent the first element of ‘admission’ or acceptance.

## VI. CONCLUSIONS

Insight has become an emblem for a rich discourse of attitudinal issues, permeating professional regulation in healthcare and used as a barometer of fitness to practise. Charting the rise and expansion of ‘insight’ in the regulation of UK doctors, this article observed early use of the term as carrying a relatively narrow, clinical meaning, reserved for cases where ‘fitness’ was being examined because of a doctor’s ill health, but steadily expanding to become a staple feature of fitness to practise decisions. As insight became embedded into the regulatory framework, the specificity of its demands increased, with successful expressions of insight tending to require a complete acceptance of responsibility^195^ for what has occurred.

These rules of professional avowal favour inward looking narratives: the doctor is the problem; they see the error of their ways; they are reformed characters or understand why their deviance or deficiency occurred and are now fully equipped to guard against recurrence. Professional norms and the reach of regulation are reaffirmed by the regulator’s action in pursuing the case, the defendant’s unqualified acceptance of the regulator’s logic, and in appeal cases, the courts’ frequent endorsement of the outcome.^196^ According to this logic, once insight is demonstrated, risk is neutralized and the public is protected for the future. On one view, the regulatory cycle (breach, enforcement, sanction, and repatriation) is complete and all is well, as long as the defendant plays their role. But on another view, the truth constituted by the avowal rests on a fiction—that we can be confident that these objective assessments of full insight, and the system of avowal, operate as a reliable proxy for risk, a fiction which is replayed in each case.

There are inevitably background risks of falsehood in the assessment of something which is so inherently subjective and this fact is no justification for seeking a substitution of insight assessment with something else. A number of specific threats to judging authenticity were explored here, with suggestions as to how they might be mitigated. Positive steps taken in this regard include the fact that through a series of ‘rejected defence’ cases, the confessional aspect of avowal has been partly detached from the curative bargain. A path is now open to doctors who persistently refuse to admit or acknowledge their wrongdoing (or propose to admit wrongdoing on their own terms), but whose acts of self-constitution in the form of commitments to regulatory requirements and doing things differently may still be accepted. Further action could be taken by extending the statutory protection of apologies to expressions of ‘insight’ to help doctors to develop insight with less fear that doing so will expose them to civil liability. Work to level out access to support and education, and to increase the diversity of tribunal membership, may be explored to ameliorate the risks of false negatives posed by any hidden curriculum of insight and cultural bias. The growing commercial market in insight training requires some oversight by regulators, and sharing intelligence between regulators on this would be beneficial if tribunals and courts are keen to distinguish between genuine and contrived expressions of insight. Finally, the rollout of ‘accepted outcomes’ to replace fitness to practise hearings in an, as yet unquantified, number of cases threatens to diminish the space for public and verbal avowals and will force increased reliance on written statements of insight. These developments should be closely monitored to gauge the gains and losses of taking this path.

All of the above signals a need for further inquiry into the effectiveness of insight as a signifier of reduced risk by examining long-term outcomes, although such research would likely only capture what might be termed ‘false positives’, that is, where a positive finding of insight was made but the doctor continued to present a risk to the public. Broader themes of inquiry should address the risks of false negatives, examining risks that findings of a lack of insight unfairly prevent good doctors returning to practice.

## Footnotes

1 For doctors, this regime is found in the Medical Act 1983 and associated rules and regulations: see Chapter 2 of J McHale and J Laing (eds), *Principles of Medical Law* (5th edn, OUP 2025) for other professions.

2 L Mulcahy and M Saks, *Regulating Health Professions* (Sage 2002) 89.

3 In the meaning used by E Pellegrino and D Thomasma, *The Virtues of Medical Practice* (OUP 2003)—‘the kind of person one is, as revealed by the virtues and vices one exhibits in one’s attitudes and actions’, at p 146.

4 The use of attitudinal issues as a reference point for assessing fitness to practise/sanction is not unique to healthcare regulation. eg for solicitors in the UK, insight clearly features in their disciplinary process, and features briefly in a handful of reported appeals, but jurisprudence is far less developed, with Justice Holroyde in *Manak v SRA* [2018] EWHC 1958 referencing the difficulties the judges were having in understanding ‘insight’ (at [45)]).

5 See ‘Regulation of Healthcare Professions’ in J. Laing and J. McHale (eds), *Principles of Medical Law* (5th Ed.) (forthcoming) at 2.07.

6 eg ss 1(1A) and 1(1B) of the Medical Act 1983 for doctors.

7 [2022] EWHC 2396 and [2022] EWHC 283, [76], respectively.

8 Acknowledged as a public good in *Bijl v General Medical Council* [2001] UKPC 42, [13]; *Bawa-Garba* [2018] EWCA Civ 1879, [93].

9 M Foucault, *Wrong-Doing, Truth-Telling: The Function of Avowal in Justice* (F Brion and BE Harcourt eds, Stephen W Sawyer tr, University of Chicago Press 2014), Lecture VI at 207, the defendant ‘punishes himself’.

10 A Tazzyman and others, ‘Reforming Regulatory Relationships: The Impact of Medical Revalidation on Doctors, Employers, and the General Medical Council in the United Kingdom’ (2019) 13 Regulation and Governance 593.

11 See R Weisman, ‘Being and Doing: The Judicial Use of Remorse to Construct Character and Community’ (2009) 18 Social and Legal Studies 47 at 51, discussed below in Section II, ‘Insight vs remorse’.

12 H Marsh, ‘The Surgeon Who Signed Patients’ Livers was Silly, not Criminal’ (*The Guardian*, 15 December 2017).

13 [2021] EWHC 2109, [50].

14 Valverde references Foucault’s ‘haunting presence’ in law and social science (M Valverde, ‘Specters of Foucault in Law and Society Scholarship’ (2010) 6 Annual Review of Law and Social Science 45 at 45.

15 Key works including but not limited to: Weisman (n 11); S Tudor and M Proeve, *Remorse: Psychological and Jurisprudential Perspectives* (Ashgate 2010); S Bandes, ‘Remorse and Criminal Justice’ (2016) 8 Emotion Review 14; S Field and T Cyrus (eds), *Criminal Justice and The Ideal Defendant in the Making of Remorse and Responsibility* (Hart Publishing 2023).

16 In Foucault's words ‘tell me who you are so that I may judge you’: Foucault (n 9).

17 ibid.

18 M Ball, ‘Review of M. Foucault, *Wrong-Doing, Truth-Telling*’ (2015) 55 British Journal of Criminal 1284, 1286.

19 I van Oorschot and others, ‘Remorse in Context(s): A Qualitative Exploration of the Negotiation of Remorse and Its Consequences’ (2017) 26 Social and Legal Studies 259, 360–61, citing M Foucault, *Discipline and Punish: The Birth of the Prison* (Pantheon, 1977).

20 ‘British doctor suspended over involvement in Just Stop Oil protests’ *The Independent*, 24 April 2024.

21 The statutory grounds for questioning fitness to practise are even broader: see, eg s 35C Medical Act 1983.

22 ss 35C and 35D Medical Act 1983. Between 200 and 300 cases are heard by tribunals annually: <https://www.gmc-uk.org/-/media/gmc-site/about/how-we-work/annual-report-2022/fitness-to-practise-annual-statistics-report-2022.pdf—table 6> accessed 8 January 2025.

23 s 35D(2) Medical Act 1983. An exception being ‘interim orders’ under s 41A where the focus is on whether restrictions are needed in advance of charges being proved.

24
*Azzam v GMC* [2008] EWHC 2711, [44].

25
*Yeong v GMC* [2009] EWHC 1923, [21].

26 ibid. s 35D(2) of the Medical Act which identifies the focus of decisions to sanction as being on whether fitness to practice ‘is’ (currently) impaired (emphasis added).

27
*Balusubramanian* [2008] EWHC 639, [10].

28
*Sanctions Guidance* (GMC, 2018, updated 2024) <https://www.gmc-uk.org/-/media/documents/DC4198_Sanctions_Guidance_Feb_2018_23008260.pdf> accessed 8 January 2025.

29
*Ibrahim v GMC* [2022] EWHC 2936, 9.

30
*El-Huseini v GMC* [2021] EWHC 2022.

31 D Lorenzini and M Tazzioli, ‘Confessional Subjects and Conducts of Non-Truth: Foucault, Fanon, and the Making of the Subject’ (2018) 35 Theory, Culture & Society 71.

32 Foucault (n 9) (also known as ‘The Louvain lectures’).

33 ibid 2.

34 ibid 209.

35 ibid.

36 Weisman (n 11) 59.

37 K Rossmanith, ‘Affect and the Judicial Assessment of Offenders: Feeling and Judging Remorse’ (2015) 21 Body & Society 167 at 176.

38 [2018] EWHC 758. Review hearing (2019) accessed via GMC register, para 22.

39 ibid.

40 J Butler, ‘Wrong-Doing, Truth-Telling: The case of Sexual Avowal’ in L Cremonesi and others (eds), *Foucault and the Making of Subjects* (Rowman and Littlefield International 2016), at 78 and 84.

41 G Pavlich, ‘Avowal and Criminal Accusations’ (2016) 27 Law Critique 229, 231–2.

42 Foucault (n 9) at 16, cited in Pavlich, ibid 231–2.

43 The rich body of remorse literature in Canada seems in part triggered by interest in the widely publicised case of Robert Latimer discussed in J Martel, ‘Remorse and the Production of Truth’ (2010) 12 Punishment & Society 414 on the Latimer case.

44
*PSA v GMC & Lingam* [2023] EWHC 967, [56].

45
*Bramhall* [2021] EWHC 2109, [48].

46 [2022] EWHC 1380, [145].

47 In *Sayer v General Osteopathic Council* [2021] EWHC 370, 25.

48 Weisman (n 11), drawing in particular from the work of Arlie Hochschild, ‘Emotion Work, Feeling Rules, and Social Structure’ (1979) 85 American Journal of Sociology 551 and (2003) and Erving Goffmann, *The Presentation of Self in Everyday Life* (Doubleday 1959).

49 Identifying 178 cases with 5+ references to remorse.

50 eg Bandes (n 15); T Maroney, ‘A Field Evolves: Introduction to the Special Section on Law and Emotion’ (2016) 8 Emotion Review 3; R Grossi, ‘Understanding Law and Emotion’ (2015) 7 Emotion Review 55.

51 A term which originated with Goffmann, albeit in a different context, but which has frequently been deployed by remorse scholars.

52 Weisman (n 11), at 49.

53 Foucault (n 9), at 209.

54 ss 40 and 40A Medical Act 1983.

55 Returned cases were manually refined to exclude cases which were not appeals/judicial review challenges of fitness to practise determinations and to include only cases where ‘insight’ was used in a fitness to practise sense as opposed to a generic, everyday sense (eg ‘Counsel were able to offer insight into how these cases are usually dealt with’).

56 1997 to the end of 2023.

57 The dip around 2020 for example, is a product of the Covid-19 pandemic which resulted in a drop in cases being processed while courts and tribunals geared up for online hearings.

58 [1997] 7 WLUK 483.

59 It is possible that the Committee’s use of insight was further developed at this time, but that it took some time for this to be more fully captured in the appeals jurisprudence.

60
*Stefan v General Medical Council* [2001] UKPC 15; *Madan v General Medical Council* [2001] EWHC Admin 577; *Hall v General Medical Council (No.1)* [2001] UKPC 46; *Whitefield v General Medical Council* [2002] UKPC 62.

61 S Gurbai and others, ‘Insight Under Scrutiny in the Court of Protection: A Case Law Survey’ (2020) 11 Frontiers in Psychiatry 1.

62 A 50:50 split between medics and lay people in the Council was not achieved until 2008 (*General Medical Council (Constitution) Order 2008* (2008/2554)).

63 P. Case, ‘Dangerous Liaisons: Psychiatry and Law in the Court of Protection.’ (2016) 24(3) Med L Rev 360.

64 ibid 367.

65 P Curk and others, ‘Removing Compliance: Interpersonal and Social Factors Affecting Insight Assessments’ (2020) 11 Frontiers in Psychiatry 1.

66
*Teewary v GMC* [2021] EWHC 376—Lack of cooperation read as failure to understand the need to cooperate with regulator’s directions and thus a failure of insight.

67 Including only a few with more than 3 x references to ‘insight’; *Pembrey; Wentzel; Gosai v General Medical Council* [2003] UKPC 31; *Qureshi v General Medical Council* [2003] UKPC 56.

68 See Fig. 1.

69 For this reason alone, quantitative measures of the use of insight do not necessarily signal deep engagement with the concept.

70 Usually these references appear because the tribunal’s perspective on the defendant’s insight (or lack thereof) is central or at least pertinent to the legal challenge.

71 Remorse literature also notes an overlapping of remorse with eg contrition (M Proeve and others, ‘Mitigation without Definition: Remorse in the Criminal Justice System’ (1999) 32 Australian and New Zealand Journal of Criminology 16 who deals with contrition and remorse as indistinct (at 17)) and with insight, and of remorse with ‘apology’ Weisman (n 11).

72 SG, Para 25 and 42–45.

73 SG, Para 25 (a).

74 SG, Para 51.

75
*Kimmance v GMC* [2016] 1808, 66.

76 See, in a similar vein to *Bramhall, PSA v GMC & Lingam* [2023] EWHC 967—lack of reasoning around the issue of insight.

77 Although the ancillary guidance for MPTs on issuing warnings does: *Guidance on Warnings* (2021), para 32, in factors to consider when deciding whether to issue a warning, includes ‘a genuine expression of regret/apology’.

78 The doctor in *GMC v Khetyar* [2018] EWHC 813, [24] had expressed ‘genuine remorse and shame’; *GMC v Bramhall* [2021] EWHC 2109, the tribunal referencing the doctor’s ‘genuine remorse’ at [10]; *Adil v GMC* [2023] EWCA Civ 1261 noting that a review tribunal would expect ‘evidence of meaningful reflection and genuine insight’ at [70]; *Garaffa v GMC* [2021] EWHC 539, [58] the MPT had noted D ‘was remorseful and genuinely regretted the incident’, to name a few.

79
*Yusuff v GMC* [2018] EWHC 13.

80
*Mmono* [2018] EWHC 3512, [12].

81 See S Maruna, *Making Good: How Ex-convicts Reform and Rebuild their Lives* (American Psychological Association 2001) at 689.

82 [2017] EWHC 2923, [27].

83 K Young and H Chimowitz, ‘How Parole Boards Judge Remorse: Relational Legal Consciousness and the Reproduction of Carceral Logic’ (2022) 56 Law and Society Review 237, 254.

84 R Zhong, ‘Judging Remorse’ (2015) 39 NYU Rev L & Soc Change 133, 172; Young and Chimowitz (above) at 240.

85 Intuition is frequently cited, and body language: L Ten Brinke, ‘Crocodile Tears: Facial, Verbal and Body Language Behaviours Associated with Genuine and Fabricated Remorse’ (2012) 21 Law and Human Behaviour 51.

86 C Berryessa, ‘Modeling “Remorse Bias” in Probation Narratives: Examining Social Cognition and Judgments of Implicit Violence during Sentencing’ (2022) 78 Journal of Social Issues 452.

87 Weisman (n 11).

88 R Zhong, ‘Judging Remorse’ (2015) 39 NYU Rev L & Soc Change 133, at 155.

89 R Zhong and others, ‘So You’re Sorry? The Role of Remorse in Criminal Law’ (2014) 42 Journal of the American Academy of Psychiatry and the Law 39, 43.

90 Weisman (n 11), at 51.

91 Young and Chimowitz (n 83) 254.

92 Gabrielle Wolf argues that ‘There are … no reliable measures to draw on to assess whether an offender is genuinely remorseful, feigning this emotion, feeling sorry for having his/her crimes detected rather than for committing them, or exhibiting some other emotion altogether’: ‘Not Black and White? Disciplinary Regulation of Doctors Convicted of Child Pornography Offences in Australia’ (2019) 45 Monash University Law Review 487, 514–5.

93 Young and Chimowitz (n 83).

94 See Foucault (n 9), at 209.

95 On ‘other regarding’ aspects of remorse see Weisman (n 11), at 58: ‘The suffering of the remorseful offender is expected to be empathic and oriented to the other rather than to the offender’s own emotional pain’.

96
*Bramhall* [2021] EWHC 2109, [48] (emphasis added). See also *Honey Rose* [2021] EWHC 2888, [73] to similar effect.

97 ibid.

98 At [62].

99 eg *Rajkumar v GMC* [2018] EWHC 1073, [14].

100 61 mentions.

101 [2022] EWHC 283, [76]. See Weisman arguing that focus on ‘cause’ of the offending is one of the features distinguishing insight from remorse: R Weisman, ‘Remorse Is Not Enough’: Disentangling the Roles of Remorse and Insight in the Construction of the Ideal Defendant’ in Stewart Field and Tata Cyrus (eds), *Criminal Justice and The Ideal Defendant in the Making of Remorse and Responsibility* (Hart Publishing 2023) 134.

102
*Khetyar v GMC* [2018] EWHC 813, [49].

103 Adapted from ‘the system’s moral logic’ Young and Chimowitz (n 83), at 251.

104 Weisman (n 11), 55.

105 Young and Chimowitz (n 83), at 255. See eg *Sengupta v GMC* [2023] EWHC 1302– blaming personal anxiety and stress caused by the GMC*; PSA v HCPC & Wood* [2019] EWHC 2819, D blaming patient for his breaching of boundaries by repeatedly initiating communications (at 74).

106
*Lateef v GMC* [2022] EWHC 2743, [23].

107
*PSA v GMC & Austin* [2023] CSIH 87, [24].

108
*Sanusi v GMC* [2019] EWCA Civ 1172, [38].

109
*Abrahaem v GMC* [2008] EWHC 183, [7].

110
*R (on the application of Baldar) v GMC* [2007] EWHC 2054.

111 [2022] EWHC 817, [76].

112 [2019] EWHC 2625, [35].

113 Dr Sarah Benn, MPTS decision 24 April 2024—<https://www.mpts-uk.org/hearings-and-decisions/tribunal-hearings-and-decisions/dr-sarah-benn—apr-24> accessed 8 January 2025.

114 Butler (n 40).

115 Broadly, the ‘attributes of a good doctor’ are defined in the GMC’s longstanding *Good Medical Practice* guidance: *Learning from Bristol: the report of the public inquiry into children’s heart surgery at the Bristol Royal Infirmary 1984 -1995* CM 5207 at 79.

116 On the ‘bad apple’ metaphor in medical regulation, see, eg K Shojania and M Dixon-Woods, ‘‘Bad apples’: Time to Redefine as a Type of Systems Problem?’ (2013) 22 BMJ Qual Saf 528.

117 R Searle and others, ‘Bad Apples? Bad Barrels? Or Bad Cellars? Antecedents and Processes of Professional Misconduct in UK Health and Social Care: Insights into sexual Misconduct and Dishonesty’ (PSA 2017).

118 R Searle and C Rice, ‘Making an Impact in Healthcare Contexts: Insights from a Mixed-Methods Study of Professional Misconduct’ (2021) 30 European Journal of Work and Organizational Psychology 470, 473.

119 eg *Simawi v GMC* [2020] EWHC 2168—taken from review hearing accessed via GMC register at [35].

120 eg *Al Nageim v GMC* [2021] EWHC 877—despite extensive insight and remediation, continued failure to show insight into one count of dishonesty resulted in erasure.

121
*Dzikowski* [2009] EWHC 1090; *Austin* [2022] CSIH 37, [24] and [26].

122
*Sanusi v GMC* [2019] EWCA Civ 1172 (tribunal at 9).

123
*Ali v GMC* [2023] EWHC 2400, [31].

124 eg see *GMC v Ahmed* [2022] EWHC 403, [39]; *Veeravalli v GMC* [2022] EWHC 747, [71]; *Motala v GMC* [2017] EWHC 2923, but there are many more.

125 eg *GMC v Mok* [2022] EWHC 165, [21].

126 J Bentley and others, ‘Apologies as Identification Management: A Theoretical Model’ (2021) 47 Public Relations 102025.

127
*GMC v Mehta* [2018] CSIH 69, [31].

128
*Mehta*, [8].

129
*PSA v GMC & Hanson* [2021] EWHC 588.

130 ibid, at [12].

131 eg *Sanusi v GMC* [2018] EWHC 1388—not appearing at a dishonesty hearing was ‘courting removal’; *Bramhall* [2021] EWHC 2109, 45 citing ‘engagement’ as an ‘attitudinal issue’; *Sherazi v GMC* [2021] EWHC 3628, non-engagement pointing towards erasure.

132 s 1(1A) Medical Act 1983.

133 V Shammas, ‘The Perils of Parole Hearings: California Lifers, Performative Disadvantage, and the Ideology of Insight’ (2019) 42 Political and Legal Anthropology Review 142, 150.

134 Resulting in a conviction for gross negligence manslaughter, albeit in a case which has prompted wide ranging reform of how such charges are dealt with: A Samanta and J Samanta, ‘Death Caused by Negligent Medical Care: Reconsidering the Role of Gross Negligence Manslaughter in the aftermath of Bawa-Garba’ (2021) 21 MLI 293.

135
*Bawa-Garba v GMC* [2018] EWCA Civ 1879, [73]. The courts disagreed with this assessment at [74].

136 See eg L Leape, ‘A Systems Analysis Approach to Medical Error’ (1997) 3 Journal of Evaluation in Clinical Practice 213.

137 [2022] EWHC 747—details of systemic factors taken from original tribunal hearing of June 2017 sourced through the GMC register.

138
*Holton v GMC* [2006] EWHC 2960, [105].

139
*Schodlok v GMC* [2015] EWCA Civ 769; also in *PSA v GMC & Uppal* [2015] EWHC 1304 the tribunal clearly referencing difficulties with senior colleagues as a possible factor in her defensive dishonesty (at [30]).

140 In *Armstrong* [2021] EWHC 1658, [56].

141
*Guidance for Chairs on Completing a Tribunal Report* DC4204, paras 8–9 (available at <https://www.mpts-uk.org/> accessed 8 January 2025).

142 Foucault appears to regard resistance as incompatible with avowal, (n 8), at 84.

143 See recommendations in *NHS Litigation Reform* HC 740 (Health and Social Care Committee, 13th Report, 2022) to explore the feasibility of a no fault model.

144 s 2 Compensation Act 2006.

145 T Wyatt and others, ‘Whispers and Shadows’: A Critical Review of the Professional Identity Literature with Respect to Minority Physicians’ (2021) 55 Med Educ 148.

146 See below at (n 164).

147 eg *Nkomo v GMC* [2019] EWHC 2625, 13—erasure for dishonesty in private life and lack of insight because specifically, D did not show acknowledgement of impact on profession of his dishonesty and conviction.

148
*Sanusi v GMC* [2018] EWHC 1388, [2].

149
*Kimmance v GMC* [2016] EWHC 1808, [66], [71].

150 See *Sengupta v GMC* [2023] EWHC 1302 a doctor’s unsuccessful attempt to challenge a finding against her on the grounds that cultural and language differences had obscured the tribunal’s assessment of her mitigation arguments.

151
*Sanctions Guidance*, paras 47, 48, and 53.

152 S Bandes, ‘Remorse, Demeanor, and the Consequences of Misinterpretation: The Limits of Law as a Window into the Soul’ (2014) 3 Journal of Law, Religion and State 170, 186.

153
*GMC v Ahmed* [2022] EWHC 403 at [73] to [74].

154
*Ahmed* [2022] EWHC 403, [77].

155 J Caballero and S Brown, ‘Engagement, not Personal Characteristics, was Associated with the Seriousness of Regulatory Adjudication Decisions about Physicians: A Cross-sectional Study’ (2019) 17 BMC Med 211.

156 ‘Overseas-trained NHS doctors more likely to be struck off without lawyer at disciplinary’ (*The Guardian* 16th May 2022). This research is based on a different time frame of hearings between 2018 and 21.

157 ‘Overseas GPs lack representation at fitness-to-practise hearings and face ‘harsher sanctions’ *Pulse*, 16th May 2022.

158
*Uwen v GMC* [2019] EWHC 3483.

159 Acknowledged in *Welcoming and Valuing International Medical Graduates* (NHS England, 2022).

160 ‘Fewer Ethnic Minority Doctors are Referred to GMC after Cases are Anonymised’ (2022) *BMJ* 377.

161 For an example of hidden curricula being applied to medical professionalism see GM Joynt and others, ‘Medical Students and Professionalism—Do the Hidden Curriculum and Current Role Models Fail our Future Doctors?’ (2017) 40 Medical Teacher 395.

162 P Jackson, *Life in Classrooms* (Holt, Rinehart & Winst 19680).

163 eg Medical Protection Society (<https://www.medicalprotection.org/> accessed 8 January 2025) or Medical Defence Union (<https://www.themdu.com/> accessed 8 January 2025).

164
*Creating a Framework for the Remediation of Unprofessional Behaviour in Medical Students*. (PhD thesis, 2021); V Goddard and S Brockbank, ‘Re-opening Pandora’s Box: Who Owns Professionalism and is it Time for a 21st Century Definition?’ (2023) 57 Medical Education 66; J Hartland, S Brockbank and V Goddard, ‘Building a Foundation of Professional Exclusion’ (2023) *BMJ Leader* Blog posted 12 June 2023 - <https://blogs.bmj.com/bmjleader/> accessed 8 January 2025.

165
*2022 at a Glance* (GMC, 2023)2022-at-a-glance-gmc-2022-annual-report_pdf-102932992.pdf (gmc-uk.org) at p 8, 5% did not specify.

166 bundle-mpts-committee-13-september-2023_pdf-103416830.pdf (mpts-uk.org) at p 11.

167
*Rezk* [2023] EWHC 3228, [59]—use of psychologist who advised writing letters to his victims which were not sent, but disclosed and accepted by tribunal as evidence of insight.

168 eg ‘Insight Works’ Training <https://insightworkstraining.co.uk/> accessed 8 January 2025; The Professional Boundaries Company <https://professionalboundaries.org.uk/courses/insight-remediation-in-practice/> accessed 8 January 2025; Probity and Ethics <https://www.probityandethics.com/> accessed 8 January 2025; Remedial Ethics <https://www.remedialethics.co.uk/> accessed 8 January 2025; HeartbeatsZAcademy <https://www.udemy.com/> accessed 8 January 2025.

169 A more effective measure (and possible vehicle for further research) would be to examine the tribunal minutes across healthcare professions. eg in *Wolverson (MPT minutes 2023)* tribunal referenced having done a course on insight run by the provider ‘Probity and Ethics’.

170
*GMC v Somuah-Boateng* [2017] EWHC 3565.

171 [2021] EWHC 1658, [54].

172
*Sawati* [2022] EWHC 283 at [39] where the absence of such courses was used to support conclusions that remediation was lacking.

173
*R v Momodou* [2004] EWCA Crim 177, [61] onwards; *Witness Preparation* (Bar Council, updated 2019), available at <https://www.barcouncilethics.co.uk/> accessed 8 January 2025.

174 R Wydick, ‘The Ethics of Witness Coaching’ (1995–1996) 17 Cardozo L Rev 1.

175 The use of coaching is not special to ftp, and was observed by Martel in the context of parole settings: J Martel, ‘Remorse and the Production of Truth’ (2010) 12 Punishment & Society 414 at 428.

176 Foucault (n 9) 17.

177 M Valverde, ‘Foucault on Avowal: Theatres of Truth from Homer to Modern Psychology’ (2015) 40 Law & Social Inquiry 1080, 1090.

178 ibid.

179 K Rossmanith, ‘Affect and the Judicial Assessment of Offenders: Feeling and Judging Remorse’ (2015) 21 Body & Society 167, 169.

180
*Yusuff v General Medical Council* [2018] EWHC 13.

181
*Yusuff* at [30].

182 <https://www.mpts-uk.org/hearings-and-decisions/hearing-types-and-how-they-work> accessed 8 January 2025.

183 Twelve months-worth of minutes appear on the MPTS webpages at <https://www.mpts-uk.org/hearings-and-decisions/medical-practitioners-tribunals> accessed 8 January 2025.

184 Subject to the GMC’s publication policy—eg that suspensions of more than 3 months disappear 15 years after expiry: *Changes to the Publication and Disclosure Policy* (Tribunal Circular, MPTS, 2018).

185
*Bramhall* [2021] EWHC 2109, [46].

186
*Anaesthesia Associates and Physician Associates Order 2024*, para 10(8).

187
*Draft—The use of accepted outcomes in fitness to practise: Guidance for regulators* (January 2024) at 7.19.

188 A request to chatGPT for a 200 word apology drafted for a doctor who administered treatment negligently and then lied about it produces a convincing response.

189 A Hochschild, *The Outsourced Self* (Metropolitan, 2013).

190 E Glikson and O Asscher, ‘AI-mediated Apology in a Multilingual Work Context: Implications for Perceived Authenticity and Willingness to Forgive’ (2023) 140 Computers in Human Behavior 107592.

191 Above at (n 142).

192 [2009] EWHC 1048, particularly [16]–[19].

193
*GMC v Awan* [2020] EWHC 1553, [35].

194
*Awan* [2020] EWHC 1553; *Sawati* [2022] EWHC 283; *Towuaghantse* [2021] EWHC 681–MPTS Review hearings retrieved via the GMC Register.

195 See text accompanying ‘the first pledge’, Part III (i).

196 Doctors appeals are rarely successful: see ‘Litigation outcomes’ in Council Meeting papers for 29 September 2022 (<https://www.gmc-uk.org/-/media/documents/council-meeting—29-september-2022—agenda-and-papers-new_pdf-96549629.pdf> accessed 8 January 2025).

